# Does Ethnic Diversity Impact on Risk Perceptions, Preparedness, and Management of Heat Waves?

**DOI:** 10.3389/fpubh.2021.642874

**Published:** 2021-08-02

**Authors:** Maya Siman-Tov, Kirsten Vanderplanken, Debarati Guha-Sapir, Joris A. F. van Loenhout, Bruria Adini

**Affiliations:** ^1^Magen David Adom, Tel Aviv, Israel; ^2^Centre for Research on the Epidemiology of Disasters, Institute of Health and Society, Université Catholique de Louvain, Brussels, Belgium; ^3^Department of Emergency Management and Disaster Medicine, Sackler Faculty of Medicine, School of Public Health, Tel Aviv University, Tel Aviv, Israel

**Keywords:** heat waves, risk perception, risk awareness, ethnic diversity, emergency preparedness

## Abstract

Detrimental health impacts of heatwaves, including excess mortality, are increasing worldwide. To assess risk perceptions, protective knowledge and behaviors concerning heatwaves in Israel, a study was initiated, comparing attitudes of majority (Jewish) and minority (Arab) populations. A quantitative survey was disseminated through an internet panel, to a representative sample of 556 individuals (79% Jews; 21% Arabs). Overall, 74% consider heatwaves a problem, 93% believe that heatwaves' frequencies will increase, 27% are very concerned about the effects of heatwaves. Higher levels of awareness to heatwaves were found among Jewish compared to Arab respondents; 90 vs. 77% (respectively) could name heatwaves' symptoms (*p* < 0.001); 81 vs. 56% (respectively) reported knowing how to protect themselves (*p* < 0.001); 74 vs. 47% (respectively) reported knowing what to do when someone suffers from heat stroke (*p* < 0.001). Arab compared to Jewish respondents presented higher levels of concern about heatwaves' effects (3.22 vs. 3.09 respectively; *t* −2.25, *p* = 0.03), while knowledge of protective measures was higher among Jews compared to Arabs (3.67 vs. 3.56 *t* = 2.13 *p* = 0.04). A crucial component of enhancing preparedness to heatwaves is empowerment of minority as well as majority groups, to strengthen their capacity to implement protective behavior and elevate their self-belief in their individual ability and fortitude.

## Introduction

Heat waves are usually defined based on their relative reference period and thresholds of very high temperatures during sustained periods of time (1–3). Despite the lack of an overall consensus on the definition of heat waves, most scientists and practitioners classify them according to their frequency, intensity, duration and timing, in relation to specific geographic areas (4, 5). Heat waves are expected to intensify, become more frequent and continue for longer durations, especially due to climate change and global warming (4, 6). Furthermore, their impact on the public health of diverse populations worldwide is anticipated to increase, even more than was seen in the past decades (1, 5).

Unless prepared for and mitigated, extreme temperatures can have detrimental health impacts, including excess mortality, even among communities that are accustomed to recurring heat waves (7, 8). In the past two decades, heat waves caused excess mortality in many countries, ranging from an increase of 38% (7, 9, 10) to more than 135% (11, 12). Sustained heat waves pose a substantial risk for vulnerable populations, including the elderly, young children, individuals that suffer from respiratory or cardiovascular diseases, as well as tourists or migrants that are not accustomed to similar weather conditions or cannot understand heat wave warnings due to language barriers (1, 13). When exposed to extreme temperature environments to which they are not physically accustomed, vulnerable populations become more susceptible to morbidity and even death (8, 14). Beyond physical morbidity and mortality, heat waves are also reported for their potentially severe impact on “mental health and societal wellbeing” (15).

Previous studies have highlighted the need to conduct multi-city investigations concerning the impact of heatwaves on varied populations (7, 16). It has been recommended that research of heat waves be conducted not only on national scales, but rather by comparing varied spatial, local and regional levels and by comparing different sectors of the population (5). This is vital as the impacts of heat waves are not derived solely from the extreme temperature but are also determined by socio-economic status, levels of emergency preparedness, and access to protective measures such as air conditioning or cooling systems (8, 17, 18).

Implementation of preparatory measures in societies contributes to a significant reduction of negative health impacts (7). Introduction of protective means is an important component of heat wave risk management, aimed to mitigate the potential risks to public health (19). National, regional and local heat wave plans and effective risk communication campaigns are aimed to enhance adaptive behavior of the population and substantially mitigate detrimental health effects (12, 20).

Ethnic differences may play a vital role in the population's risk perceptions and capacity to adapt their behavior to effectively manage the consequences of heat waves (21, 22). Studies have presented that varied ethnic affiliations may be related to a higher vulnerability to heat waves (resulting from socio-economic and cultural-behavioral differences), such as among African American populations in the US or indigenous Australians, resulting in increased rates of morbidity and mortality (21, 23). As ethnicity may impact on both risk perception as well as coping behaviors, there is a need to study the effects of ethnicity on heat wave vulnerability for majority and minority populations (12, 22).

Reducing health inequalities is one of the Strategic Development Goals (SDGs) adopted by the international community, which directly relates to accessibility of all ethnicities to health services. The Israeli society consists of diverse ethnicities, among which almost 80% of the population are Jewish and 20% are non-Jews, consisting of Muslim, Bedouin and Christian Arabs. Arab Israelis vs. Jewish Israelis are considered two different ethnicities, due to variance concerning the religious, cultural, language, norms, values as well as tradition diversities. Despite the universal health coverage that entitles all residents to access a basic basket of medical services, inequities in risk perceptions, varied levels of trust in the medical systems and dissimilar degrees of accessibility to healthcare services were reported (24, 25).

Considering the frequent heat waves that occur in the Middle East, their impact on varied sectors of the population, the potential diversity in their risk perceptions and adoption of protective measures, a study was implemented, designed to assess the risk perception, protective knowledge and behavior concerning heat waves in three geographic locations in Israel, comparing the views and attitudes of both majority (Jewish Israeli) and minority (Arab Israeli) populations. The study was conducted as part of a European consortium—the EC-funded SCORCH project (grant agreement no. 826565), designed to foster a cross-country culture of preparedness, prevention, mitigation and cooperation regarding heat waves.

## Materials and Methods

### Participants

The study was conducted among a sample of the Israeli population, representing the overall proportion of the two main ethnicities, in three specific locations. According to calculation of OpenEpi (https://www.openepi.com), 385 respondents were required, of which 80% Jews and 20% Arabs (the two main ethnicities). A stratified sampling method was used to achieve a representative sample, based on data published by the Israeli Central Bureau of Statistics in regard to age, ethnicity, religiosity, socio-economic level, and geographic zones. The internet panel employed in the study filtered the target population, in accordance to the above-mentioned categories. The sample population was selected from three major urban communities, each located in a different geographic location (the northern, central and southern regions) to encompass three areas that represent varied climatologic characteristics.

### Study Tool

The study was based on a structured quantitative survey that was developed and validated in previous studies (26, 27). The survey contained five main sections namely demographics; awareness of heat waves; health impacts; knowledge and behaviors aimed at reducing health impacts; and, protective measures for decreasing their impacts.

The questions on awareness and knowledge and behavior were further classified to six indices as follows: (1) awareness about health impacts of heatwaves (10 items, Cronbach α = 0.872); (2) recognition of groups vulnerable to health effects due to heatwaves (13 items, Cronbach α = 0.854); (3) level of concern about effects of heatwaves on oneself or others (9 items, Cronbach α = 0.816); (4) knowledge about protective measures to reduce health impacts of heatwaves (7 items, Cronbach α = 0.715); (5) familiarity with self-protective measures (12 items, Cronbach α = 0.804); and, (6) actions implemented during the last heatwave (12 items, Cronbach α = 0.856). Concerning each of the five initial indices, the respondents were asked to indicate through a dichotomous yes/no question if they are knowledgeable and indicate on a 3-level scale how concerned they were (ranging from not at all, through somewhat concerned to very concerned). Following each answer, they were presented with the detailed items, and requested to mark next to each item their level of knowledge /concern respectively on a four-point scale. In addition, respondents had the option to answer “I do not know” to each question; such an indication was calculated as missing data when creating the six indices. A yes/no question did not precede the sixth index; the respondents were requested to indicate the extent of their implementation of each protective measure.

To ensure both content quality, validity and reliability of the survey, it was translated from English to Hebrew as well as Arabic, and re-translated to English The survey was pilot tested among a sample of both Hebrew and Arabic speaking individuals (*n* = 30 and 20, respectively), prior to its dissemination to the full target population.

### Study Design

The survey was disseminated by an internet panel company, that consists of over 100,000 panelists, representing all geographic and demographic sectors of Israel (http://www.ipanel.co.il/). The Jewish respondents received the survey in Hebrew while the Arab respondents received it in both Arabic and Hebrew. The survey was approved by the Ethics Committee of the Tel Aviv University, number 0000016-1 from July 16, 2019.

### Statistical Analysis

Descriptive statistics were used to describe the characteristics of the sample. Sociodemographic characteristic differences between the two population groups were tested by chi square test (for categorical variables) and independent samples *t* test (for numerical variables). Different perceptions on heat waves were analyzed using chi square tests followed by multiple logistic regressions, adjusted for age and gender. Differences concerning information sources for accessing data were examined using chi square test. All statistical analyses were performed using SPSS software version 25. *P*-values lower than 0.05 were considered to be statistically significant, based on two-sided tests.

## Results

The respondents comprised a total of 556 individuals, among them 79% (*N* = 438) Jews and 21% (*N* = 118) Arabs. The sociodemographic characteristics of the study population in the total sample and according to each specific ethnic population group is presented in Table 1.

**Table 1:** Demographic characteristics of the respondents by population group.

**Characteristics**	**Total *n* = 556**	**Jews *n* = 438**	**Arabs *n* = 118**	***P*-value**
Gender				
Male	36.4%	42.6%	13.6%	<0.001
Age				
18–21^*^	10.8%	10.0%	13.5%	<0.001
22–40	56.1%	50.3%	78.0%	
41–60	24.6%	29.0%	8.5%	
61+	8.5%	10.7%	0	
Education				
Up to and including high school	31.6%	36.1%	14.7%	<0.001
Vocational	15.4%	15.2%	16.4%	
Academic	53.3%	48.7%	69.0%	
Religiosity				
Not religious	54.3%	56.9%	44.3%	0.001
Slightly religious	17.7%	16.4%	22.6%	
Moderately religious	21.4%	18.8%	31.3%	
Very religious	6.6%	7.9%	1.7%	
Children below 12	32.7%	33.1%	31.4%	0.719
Employment status				
Student	12.8%	9.4%	25.4%	< .001
Employed	71.6%	73.5%	64.4%	
Unemployed	4.5%	3.7%	7.6%	
Retired	4.1%	5.3%	0	
Other	7.0%	8.2%	2.5%	
Income level				
Less than average	39.0%	34.3%	54.9%	<0.001
Average	29.3%	29.1%	34.1%	
Above average	31.7%	36.6%	15.0%	
Mainly outdoors work	26.6%	23.3%	40.8%	0.002
Working taking care of others	21.6%	34.2%	18.6%	0.003
Fasted in the past 5 months	26.3%	23.5%	36.4%	0.005
Consume medications for chronic disease	21.4%	24.4%	10.2%	0.001

* *This age group was chosen to differentiate pre-post mandatory conscription age*.

Approximately 57.4% of the Jewish sample were female compared to 86.4% among the Arab sample (*p* < 0.001). The average age of the respondents was 36.3; Arabs compared to Jews were younger (28.5 vs. 38.4; *p* < 0.001), had higher levels of academic education (69 vs. 49%; *p* < 0.001), more were students (25 vs. 9%; *p* < 0.001), worked more often outdoors (41 vs. 23%; *p* = 0.002), reported on higher rates of participating in fasting activities (36 vs. 24%; *p* = 0.005) and, had lower levels of consuming medications for chronic diseases (10 vs. 24%; *p* = 0.001).

Among the total sample of the respondents that stated their view (yes or no), 74% consider heatwaves as a problem, 93% believe that heatwaves will occur more frequently in the future, and approximately 27% feel very concerned about the effects of heatwaves on themselves or on others. No significant differences were found between Arab and Jewish respondents.

**Figure 1:**
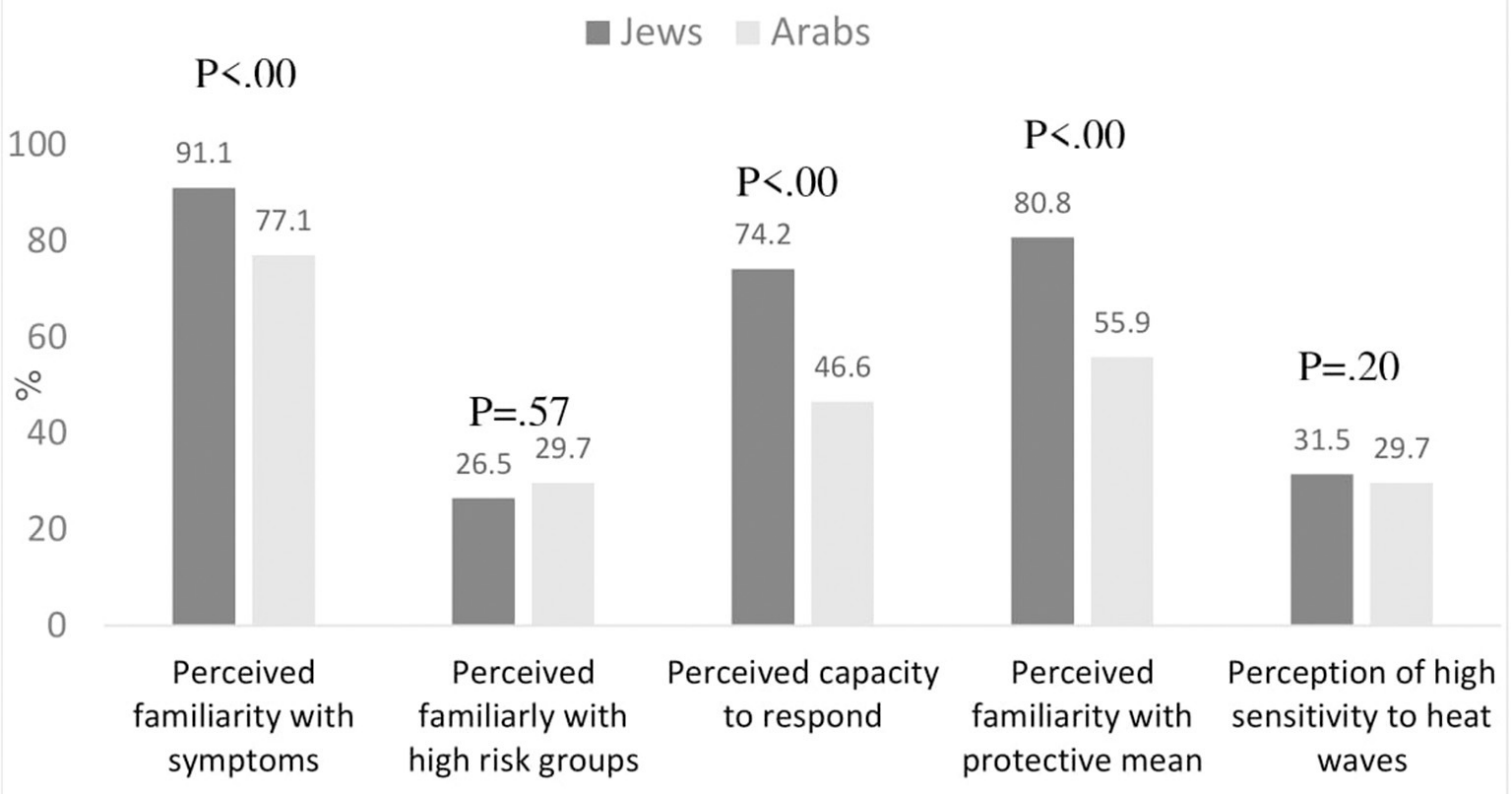
Levels of awareness concerning heat waves among Jewish and Arab respondents.

Overall, significantly higher levels of perceived awareness concerning varied aspects related to heat waves were found among the Jewish compared to the Arab respondents (Figure 1).

Nearly 90% of the Jewish sample reported that they could name some of the symptoms due to heat waves, compared to 77% among the Arab sample (*p* < 0.001). Approximately 81% of the Jewish sample reported knowing how to protect themselves against heat waves, compared to 56% among the Arab sample (*p* < 0.001), and 74% of the Jewish sample reported knowing what to do when someone suffers from a heat stroke, compared to 47% of the Arab sample (*p* < 0.001). A relatively high percentage (~87%) of both Jewish and Arab respondents reported recognition of groups at risk from heat waves. In addition, 30% reported that they define themselves as highly sensitive to heat, with no significant difference between Jewish and Arab respondents.

**Figure 2:**
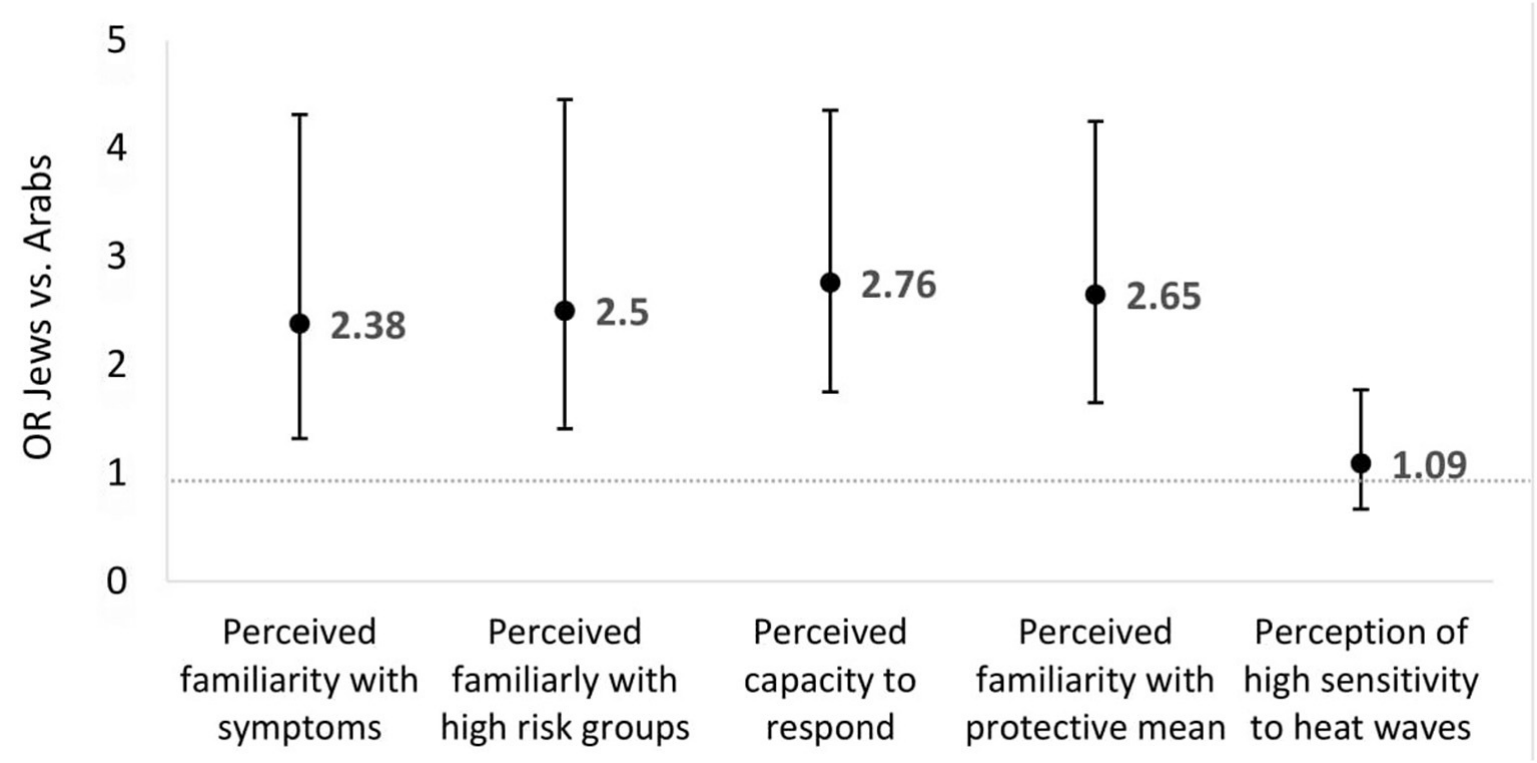
Regression models of perceived familiarity of heat waves among Jewish vs. Arab respondents.

Due to the significant differences between the two population groups (Jewish and Arab respondents) in gender composition, age, educational and occupational status, a logistic regression was performed to predict their perceptions/ knowledge regarding heat waves after adjusting for the diverse sociodemographic variables. The results of the regression models are presented in Figure 2.

Based on Multiple logistic regression adjusted to age and gender.

Data for Figure 2:

- Perceived familiarity with symptoms Jews/Arabs OR 2.38 95CI 1.32–4.31; *p* = 0.004.
- Perceived familiarly with high risk groups Jews/Arabs OR 2.50 95%CI 1.41–4.45; *p* = 0.002.
- Perceived capacity to respond Jews/Arabs OR 2.76 95%CI 1.75–4.35; *p* < 0.001.
- Perceived familiarity with protective mean Jews/Arabs OR 2.65 95%CI 1.65–4.25; *p* < 0.001.
- Perception of high sensitivity Jews/Arabs OR 1.09 95%CI 0.67–1.77; *p* > 0.05.

As is clearly visible, the Jewish respondents more often indicate that they are knowledgeable of the varied consequences of heat waves compared to the Arab respondents, including in recognizing symptoms, identifying risk groups, knowing what to do when someone suffers from heat stroke and proficiency concerning protective means, even after controlling for sociodemographic characteristics.

When comparing the responses of the Arab and the Jewish respondents for the six indices (namely aware of health impacts, recognize vulnerable groups, levels of concern, know about protective measures, familiar with self-protective measures, and implement actions), significant differences were found only for the levels of concern about the heat waves' effects. Arab compared to Jewish respondents presented a significantly higher level of concern (3.22 vs. 3.09 respectively; *t* −2.25, *p* = 0.03). In contrast, knowledge of protecting measures was higher among Jews compared to Arabs (3.67 vs. 3.56 *t* = 2.13 *p* = 0.04). Concerning the other four indices, no significant differences were found between the two population groups. See Table 2.

**Table 2:** Index differences between population groups.

**Index**	**Jews**	**Arabs**	***t***	***P*-value**
Awareness of health impacts of heatwaves (signs and symptoms)	3.36 ± 0.54	3.34 ± 0.63	−0.46	0.65
Awareness of risk groups	3.29 ± 0.45	3.31 ± 0.56	−0.49	0.62
Level of concern about effects of heatwaves	3.09 ± 0.55	3.22 ± 0.59	−2.25	0.03
Knowledge of protecting measures	3.67 ± 0.36	3.56 ± 0.51	2.13	0.04
Familiarity with self-protection measures	3.26 ± 0.41	3.29 ± 0.50	−0.061	0.54
Personal protection actions implemented during the last heatwave	2.63 ± 0.57	2.73 ± 0.64	−1.59	0.11

The sources of information concerning heat waves that were reported by the respondents are presented in Table 3.

**Table 3:** Differences in information resources.

**Characteristics**	**Jews *n* = 438**	**Arabs *n* = 118**	***P*-value**
**Informed about heatwaves**			
Very well-informed	9.4%	11.0%	0.059
Somewhat informed	48.6%	38.1%	
Not very well-informed	34.5%	36.4%	
Not at all informed	7.5%	14.4%	
**Looking for information last heat wave from;**			
Government social media or website	27.2%	35.6%	0.073
Government brochure or poster	1.4%	4.2%	0.061
Television	39.5%	33.9%	0.267
Radio	15.1%	16.1%	0.782
Newspaper or news website	34.7%	29.7%	0.303
Doctor or health professional	1.4%	4.2%	0.061
Relatives or friends	10.0%	20.3%	0.002
Not consulted any source	33.1%	28.0%	0.288
Prefer to be notified about heatwave occurrence	72.4%	76.3%	0.594
**Last summer consulted with:**			
Government social media or website on measures	24.7%	39.0%	0.002
Government brochure or poster on measures	1.6%	5.1%	0.038
Television on measures	39.7%	36.4%	0.516
Radio on measures	18.3%	18.6%	0.925
Newspaper or news website on measures	31.7%	31.4%	0.937
Doctor or health professional on measures	3.7%	5.1%	0.479
Relatives or friends on measures	11.9%	22.0%	0.005
Did not consult a source on measures	39.0%	32.2%	0.173
**Expect to get information from:**			
Police	36.6%	47.6%	0.068
Fire brigade	75.6%	72.9%	0.591
Mayor	54.0%	63.4%	0.100
Media	92.4%	93.0%	0.636
Religious leaders	6.9%	17.7%	0.006
Health care professionals	89.1%	89.8%	0.826
Social services	64.5%	65.6%	0.840
Care institutions	50.1%	61.1%	0.059
Relatives or friends	59.4%	75.5%	0.003
Community leaders	33.3%	58.4%	<0.001

Approximately half of the sample reported being informed about heat waves, although almost 75% reported that they prefer to be personally notified about heat wave occurrence. The most common source that is utilized by both Jews and Arabs to search for information on heat waves is the television (39.5 vs. 33.9%, respectively), closely followed by newspapers or news sites (~34.7 vs. 29.7%, respectively) and governmental social media or website (27.2 vs. 35.6% respectively). In contrast, survey participants reported that they rarely search for information through brochures or posters from government offices (only 3%), their physician or other medical professionals (about 3%). Arab vs. Jewish respondents more frequently (and significantly) prefer to access information from religious leaders, relatives, friends or community leaders.

## Discussion

Heat waves occur frequently in the Middle East countries, including in Israel, and present a health risk (28). As there are substantial potential detrimental impacts of heat waves on the population (7, 8), most especially on vulnerable groups (1, 13), it is vital to ensure that all individuals are aware of the hazards, understand the potential impact, and adopt protective behaviors that have proven to be effective in mitigating the consequences (20). Identifying the perceptions, attitudes and behavior of the varied groups in the population concerning heat waves, should be of high priority for policy-makers and governance systems. Having insights on population behavior and attitudes can tailor risk communication strategies and information directed to the public to the needs and characteristics of the varied population groups (12, 29). Campaigns for health protection against heatwaves have to consider psycho-social and cultural characteristics of different ethnicities, as these influence both the perception of the risk and its consequences, as well as the willingness to adopt and implement practical steps in view of the risk (28, 29).

Our study found that the overall level of awareness among the Israeli population to the risk of heat wave, their potential impacts, as well as means to protect oneself from negative outcomes is relatively high (90%), compared to other studies conducted in countries that are routinely accustomed to higher temperatures (such as Spain and Portugal) vs. moderate temperatures (Netherlands and Belgium) (26, 27).

As heat waves do not discriminate between populations and the extreme temperatures are equally felt, it may have been expected that familiarity with signs, symptoms and protective measures would be similar among all populations (30, 31). Conversely, some previous studies suggest that minority populations (such as Hispanics or African Americans in the US or Turks in Austria) are often more concerned and vulnerable than the majority groups in regard to climate change and heatwaves (23, 28, 30). The current study's results may shed some light on the variability found between minority to majority populations. Initially we found that both ethnic groups (Arabs and Jews) have similar levels of concern and similarly believe that heatwaves will increase in the future. When asked directly about their levels of knowledge concerning heatwaves, Arab respondents answered less often as being knowledgeable than the Jewish respondents. Nonetheless, when faced with a list of signs and symptoms or measures that may reduce the health impacts of extreme temperatures, no differences were found between the two groups. This variability concerning the perceived knowledge rather than actual proficiency of minority vs. majority ethnic groups is aligned with the contention that ethnic minorities do not attribute to themselves leadership or guidance capacities (29, 32). The lower levels of perceived knowledge, awareness and capacity to effectively respond to heat waves among minority populations, justifies more in-depth understanding.

Furthermore, the differences that were found between the two populations in regard to levels of awareness of vulnerable groups (though not found significant), may be attributed to varied cultural and occupational diversities (23, 30, 31). Similar to the challenges faced by other minorities, Arabs compared to Jews have lower socio-economic levels and a higher percentage of working outdoors (23–25). Both characteristics may lead to a higher exposure to impacts of heat waves, with a lower capacity to mitigate its consequences through air conditioners or other protective measures (8, 17, 18, 33).

Higher levels of awareness among the Arab respondents to vulnerable populations may be derived from the family structures among this group. It is customary for Arab families of several generations (children, parents, grandparents and even great-grandparents) to reside together, thus enhancing their sensitivity to the diverse needs of both children and the elderly, including when they are suffering from varied illnesses (34, 35).

These social and cultural differences may also explain the variance concerning sources for accessing reliable information concerning the risk and its consequences (23, 29). Both Arab and Jewish populations search for relevant data through governmental social media or websites, as well as via the television or radio. Nonetheless, more than twice the extent of Arab compared to Jewish respondents approach their relatives and friends in order to access such vital information (35). Furthermore, a higher percentage of Arab vs. Jewish respondents expect to be informed about heat waves by their religious prominent figures or their community leaders. It seems their community ties and segregation are stronger and they attribute a higher level of trust to their in-group resources (32, 34).

Finally, most respondents, for both study sub populations prefer to be personally notified about a forecasted occurrence of a heat wave rather than access the information independently, using their own resources. It could be expected that due to the abundant influx of data, forecasting services that are accessible, and extensive media coverage, that individuals would perceive the information as easily accessible and available (4, 7). This need to be personally informed may be derived from the concern of many that due to the routine high temperatures that characterize the Middle East region, they will remain unaware of the need to implement additional designated measures during extreme heat waves that are vital to facilitate their safety and well-being (24, 25). It may also be related to the high digital literacy of all population groups in Israel in utilizing WhatsApp, which is a perceived by the individuals as being “personally informed” (36).

We faced limitations in the implementation of this study. The first is the use of an internet panel to recruit respondents. Some doubt may be cast as to the level of representation of the respondents and their similarity to the overall target population (37). In order to mitigate this risk, the study was conducted through the largest internet panel that operates in Israel, consisting of over 100,000 panelists that represent the diverse groups in the population, with a high influx of new participants and strict quality assurance measures. The second is the variance of characteristics among the two main population groups. This was corrected by using logistic regression models after adjusting for the differences that were found between the Arab vs. the Jewish population groups. The third is the use of some closed-answer questions that may not reflect the actual knowledge of the respondents but rather what they believe are socially desirable answers.

## Conclusions

Heatwaves pose a significant risk to the health and well-being of the global community and are expected to increase in both frequency and intensity due to global warming and climate change. The capacity to respond effectively to the detrimental impact of heat waves is dependent on the risk perception, awareness and behavior of the public to a large extent. It is thus vital to understand the variability that exists among different ethnicities concerning heatwaves and tailor the national and local risk communication strategies to the specific needs of the different population groups. A crucial component of enhancing preparedness to heatwaves is empowerment of all sections of the population, minority as well as majority groups that can strengthen their capacity to implement protective behavior and elevate their self-belief in their individual ability and fortitude. It is recommended that future studies be implemented which investigate the impact of different communication programs on perceived risks, awareness and protective behavior of different ethnicities, to facilitate the implementation of effective risk management tools.

## Data Availability Statement

The raw data supporting the conclusions of this article will be made available by the authors, without undue reservation.

## Ethics Statement

The studies involving human participants were reviewed and approved by Institutional Ethics Committee of the Tel Aviv University. The patients/participants provided their written informed consent to participate in this study.

## Author Contributions

DG-S, JvL, and BA: conceptualization. BA and KV: methodology. BA, JvL, DG-S, and KV: validation. MS-T: formal analysis. BA: investigation. BA and MS-T: writing—original draft preparation. DG-S, JvL, and KV: writing—review and editing. All authors have read and agreed to the published version of the manuscript.

## Conflict of Interest

The authors declare that the research was conducted in the absence of any commercial or financial relationships that could be construed as a potential conflict of interest.

## Publisher's Note

All claims expressed in this article are solely those of the authors and do not necessarily represent those of their affiliated organizations, or those of the publisher, the editors and the reviewers. Any product that may be evaluated in this article, or claim that may be made by its manufacturer, is not guaranteed or endorsed by the publisher.
