# Supplementary material for: Does Ethnic Diversity Impact on Risk Perceptions, Preparedness, and Management of Heat Waves?
Source: Front Public Health. 2021 Aug 2;9:642874. doi: 10.3389/fpubh.2021.642874 (PMC8365166; doi:10.3389/fpubh.2021.642874)
Supplement: Supplementary file 1 [file Table_1.DOCX]

**Online questionnaire**

**introduction**

**Introduction and Request for Consent (see separate file)**

YES  NO 🡺 If not, is not suitable for this study

1. **Inclusion Criteria**
2. Do you currently live in the city / country of relevance to the research?

YES  NO 🡺 If not, is not suitable for this study

🡺 If yes: Have you lived here for more than 5 years?

YES  NO

1. What is your age?

If under the age of 18 🡺 not suitable for this study

1. **Awareness of formation of heat waves**
2. Do you know when the last heat wave occurred in your city??

No  Yes, Year _________

Do you consider heat waves in your city as a problem?

No  YES  don’t know

1. Do you believe that in the future to come, heat waves will occur more often in your city?
2. No  Yes  don’t know
3. **Awareness about the effects of heat waves on health**
4. a) Can you list some symptoms that people may experience as a result of heat waves?

No  Yes: (Include "Add Answer" option to have only one answer per line)

b) On a scale from "Absolutely not" to "Absolutely", what symptoms do you think are related to heat waves?

|  | Absolutely not | Probably not | Probably | Absolutely | Don’t Know |
| --- | --- | --- | --- | --- | --- |
| Headache |  |  |  |  |  |
| Dehydration |  |  |  |  |  |
| exhaustion |  |  |  |  |  |
| Extreme thirst |  |  |  |  |  |
| Nausea / vomiting |  |  |  |  |  |
| Muscle spasm |  |  |  |  |  |
| Disorientation |  |  |  |  |  |
| interrupted Sleep |  |  |  |  |  |
| diarrhoea |  |  |  |  |  |
| Difficulty breathing (hyperventilation) |  |  |  |  |  |

1. a) Can you list groups of people at higher risk of health effects from heat waves?

Don’t know  Yes: Include "Add Answer" option to have only one answer per line")

b) To what extent do you consider the following groups to be vulnerable to the effects of heat waves on health?

|  | Not vulnerable at all | Not very vulnerable | vulnerable | Very vulnerable | Don’t Know |
| --- | --- | --- | --- | --- | --- |
| Elderly |  |  |  |  |  |
| Infants and children under the age of 12 |  |  |  |  |  |
| Pregnant women |  |  |  |  |  |
| People who take medication every day |  |  |  |  |  |
| People with physical illness |  |  |  |  |  |
| People with mental illness |  |  |  |  |  |
| People with physical disabilities or people with mobility problems |  |  |  |  |  |
| People living in cities |  |  |  |  |  |
| People living in poor housing conditions (eg, poor insulation) |  |  |  |  |  |
| People are socially isolated |  |  |  |  |  |
| Homeless people |  |  |  |  |  |
| Physically active people |  |  |  |  |  |
| People who fast |  |  |  |  |  |

1. How concerned are you about the effects of heat waves on you or on others?

**If you are not worried at all, go to Question No. 9**

Somewhat worried

Very worried

**🡺 If you are somewhat worried or very worried, what or who worries you and to what extent?**

|  | Not worried at all | Not very worried | Worried | Very worried | I don’t know |
| --- | --- | --- | --- | --- | --- |
| My personal health |  |  |  |  |  |
| Health of my child/children |  |  |  |  |  |
| Health of elderly relatives |  |  |  |  |  |
| The health of my neighbours or people in my community |  |  |  |  |  |
| Financial Aspects |  |  |  |  |  |
| Temperature at school or at work |  |  |  |  |  |
| The health of my pets |  |  |  |  |  |
| My participation in outdoor activities |  |  |  |  |  |

other:

1. **Knowledge about and behaviour to reduce the effects of heat waves on health**
2. a) Do you know what to do when someone has a heat stroke/overheating? (You can answer more than one answer)

No  Yes (Include "Add answer" option, to have only one answer per line)

b) What steps do you consider to be effective in helping a person suffering from a heat wave?

|  | Not at all effective | Very little effective | effective | Very effective | Don’t know |
| --- | --- | --- | --- | --- | --- |
| Halt his/her physical activities |  |  |  |  |  |
| Drinking |  |  |  |  |  |
| Place the person in a cool location |  |  |  |  |  |
| Call for emergency services |  |  |  |  |  |
| Remove warm clothing |  |  |  |  |  |
| Place the person in a shower or sprinkle with lukewarm water |  |  |  |  |  |
| Give the person medication to lower body temperature |  |  |  |  |  |

1. a) **Do you know steps that you can take to protect yourself from a heat wave?**

No  Yes: (include "Add answer" option to have only one answer per line)

b) What steps do you consider to be effective in protect yourself or others from a heat wave?

|  | Not effective at all | Not very effective | effective | Very effective | Don’t know |
| --- | --- | --- | --- | --- | --- |
| Drinking more water |  |  |  |  |  |
| Stay indoors during the hottest hours |  |  |  |  |  |
| Stay in a park or in a green area |  |  |  |  |  |
| Wear light clothing in light colors |  |  |  |  |  |
| Cool my body with a shower |  |  |  |  |  |
| Go swimming |  |  |  |  |  |
| Consult my doctor about my medication |  |  |  |  |  |
| Use a fan or air conditioner |  |  |  |  |  |
| Taking medication to lower my body temperature |  |  |  |  |  |
| Wearing a hat |  |  |  |  |  |
| Limiting physical activity |  |  |  |  |  |
| Close the windows and curtains |  |  |  |  |  |
| Eat light meals |  |  |  |  |  |

1. **Do you consider yourself sensitive to heat?**

Very  Somewhat  Not at all

1. **In the last heat wave, indicate how much you**

|  | Always | Usually | Sometimes | Never | Not relevant |
| --- | --- | --- | --- | --- | --- |
| drank more water |  |  |  |  |  |
| Stayed inside during the hottest hours |  |  |  |  |  |
| Stayed in a park or a green area |  |  |  |  |  |
| Wore light clothing in light colors |  |  |  |  |  |
| Cooled your body with a shower or swimming |  |  |  |  |  |
| Adjusted your medications |  |  |  |  |  |
| Used a fan or an air conditioner |  |  |  |  |  |
| Took medication to lower your body temperature |  |  |  |  |  |
| Wore a hat |  |  |  |  |  |
| Limited physical activity |  |  |  |  |  |
| Closed windows and curtains |  |  |  |  |  |
| Ate light meals |  |  |  |  |  |

1. **A. Sources of information on heat waves and protection measures:**
2. Do you consider yourself informed about heatwaves?

Very informed

Somewhat informed

Not very well informed

Not informed at all

1. During the last heat wave, where were you looking for information about the weather? (You can choose multiple answers)

Government social media or website

Government brochure or poster

TV

Radio

Newspaper or online news website

Doctor or other health professional

Relatives / Friends

I did not consult any source of information about protective measures

Other:

1. Did the government or other organization inform you personally about a heat wave?

No  I don’t know

Yes, this source

Via (e-mail, phone, ......)

1. Would you prefer to be notified of a future heat wave?

No  Yes  I don’t know

1. Which of the following information sources did you use last summer to search for information about steps to protect yourself from the heat wave? (More than one answer can be selected)

Government social media or website

Government brochure or poster

TV

Radio

Newspaper or online news website

Doctor or other health professional

Relatives / Friends

I did not consult any source of information about protective measures

Other:

1. From which of the following bodies do you expect to receive information about what to do during the next heat wave?

|  | Yes | No | Not relevant |
| --- | --- | --- | --- |
| Police |  |  |  |
| Fire brigade |  |  |  |
| Mayor |  |  |  |
| Media (press) |  |  |  |
| Religious leaders |  |  |  |
| Health professionals |  |  |  |
| Social services |  |  |  |
| Care institutions |  |  |  |
| Family and friends |  |  |  |
| Community leaders |  |  |  |

Other:

1. **Demography**
2. Gender

Male  Female  Other

1. What is your level of education?

Not a graduate of the education system

Graduated from elementary school

High school graduate

Graduate of a vocational school or diploma studies

Graduated from college or university

1. Do you have children 12 years or under?

No  Yes

1. What is your current employment status?

Student 🡺 Skip to Question 23

Employee or self-employed

Unemployed **🡺** Skip to Question 23

Pensioner 🡺 Skip to Question 23

Other **🡺** Skip to Question 23

**🡺** If an employee or self-employed: Does your work require you to work primarily outside between 10:00 and 15:00.

No  Yes

🡺 If you are an employee or self-employed: Does your work include taking care of other people (e.g., in a kindergarten, school, or care centres for the elderly)?

No  YES

1. Do you care for a relative who is dependent on you? (Such as a child, a relative with a disability, an elderly relative)

No  Yes

1. Have you taken part in several days of fasting during May to September (such as fasting for religious reasons)?

No  Yes

1. Do you take medications every day for a chronic condition? (Such as high blood pressure, diabetes, asthma)

No  Yes

1. To what extent do you consider yourself religious?

not religious

A bit religious

Religious to a moderate extent

Very religious

don’t know

1. The average monthly gross income per employee today is NIS 10,800. In relation to this, what is the income level of your family?

Less than the average

Average

More than average

Refuse to answer

**Summary**

Thank you very much for your participation. If you would like to have a brief summary of our findings, you can provide us with your email address. This email will not be used for any other purpose.

Do you have any other feedback for the researchers?
